# On the Design of an Efficient Cardiac Health Monitoring System Through Combined Analysis of ECG and SCG Signals

**DOI:** 10.3390/s18020379

**Published:** 2018-01-28

**Authors:** Prasan Kumar Sahoo, Hiren Kumar Thakkar, Wen-Yen Lin, Po-Cheng Chang, Ming-Yih Lee

**Affiliations:** 1 Department of Computer Science and Information Engineering, Chang Gung University, Guishan 33302, Taiwan; pksahoo@mail.cgu.edu.tw (P.K.S.); D0221015@cgu.edu.tw (H.K.T.); 2 Department of Electrical Engineering and Center for Biomedical Engineering/College of Engineering, Chang Gung University, Guishan 33302, Taiwan; wylin@mail.cgu.edu.tw; 3 Graduate Institute of Medical Mechatronics, Center for Biomedical Engineering, Chang Gung University, Guishan 33302, Taiwan; 4 Division of Cardiology, Department of Internal Medicine, Chang Gung Memorial Hospital, Linkou 33305, Taiwan; pccbrian@gmail.com

**Keywords:** cardiovascular disease (CVD), electrocardiogram (ECG), seismocardiogram (SCG), cardiac anomalies

## Abstract

Cardiovascular disease (CVD) is a major public concern and socioeconomic problem across the globe. The popular high-end cardiac health monitoring systems such as magnetic resonance imaging (MRI), computerized tomography scan (CT scan), and echocardiography (Echo) are highly expensive and do not support long-term continuous monitoring of patients without disrupting their activities of daily living (ADL). In this paper, the continuous and non-invasive cardiac health monitoring using unobtrusive sensors is explored aiming to provide a feasible and low-cost alternative to foresee possible cardiac anomalies in an early stage. It is learned that cardiac health monitoring based on sole usage of electrocardiogram (ECG) signals may not provide powerful insights as ECG provides shallow information on various cardiac activities in the form of electrical impulses only. Hence, a novel low-cost, non-invasive seismocardiogram (SCG) signal along with ECG signals are jointly investigated for the robust cardiac health monitoring. For this purpose, the in-laboratory data collection model is designed for simultaneous acquisition of ECG and SCG signals followed by mechanisms for the automatic delineation of relevant feature points in acquired ECG and SCG signals. In addition, separate feature points based novel approach is adopted to distinguish between normal and abnormal morphology in each ECG and SCG cardiac cycle. Finally, a combined analysis of ECG and SCG is carried out by designing a Naïve Bayes conditional probability model. Experiments on Institutional Review Board (IRB) approved licensed ECG/SCG signals acquired from real subjects containing 12,000 cardiac cycles show that the proposed feature point delineation mechanisms and abnormal morphology detection methods consistently perform well and give promising results. In addition, experimental results show that the combined analysis of ECG and SCG signals provide more reliable cardiac health monitoring compared to the standalone use of ECG and SCG.

## 1. Introduction

Recent advancements in sensor technology have made it possible to use low-powered, inexpensive sensor-based devices to monitor various physiological parameters related to human health such as heart rate, blood pressure, body temperature etc., [1,2]. Numerous applications are becoming reality in the light of wearable sensor technology such as diet monitoring, drug monitoring, activity detection, cardiac health monitoring, etc. [3]. Among the envisioned applications, designing a robust cardiac health monitoring system to capture early signs of gradually developing cardiac anomalies using low-cost wearable body sensors is a growing interest among medical and research communities as it has serious consequences on human health. According to World Health Organization [4], CVD is the leading cause of death in people across all age groups. Moreover, the recent report of the American Heart Association [5] reveals that as high as 30% annual mortality rate is observed due to CVD in the United States and Canada. Under such circumstances, there is a growing need for a reliable and low-cost system that potentially aids in robust cardiac health monitoring.

Several cardiac events take place during successive heartbeats such as opening and closing of the heart valves, blood flow into vessels, contraction–relaxation of ventricular walls, etc. For a healthy person, cardiac events unfold at regular intervals in a predefined order. However, cardiac abnormalities such as myocardial ischemia, infarction, arrhythmias, etc., hinder the normal functioning of cardiac events leading to CVD. Such cardiac abnormalities may result in dizziness, nausea, chest pain, etc., which may lead to severe consequences such as heart attacks if not detected and taken care of in an early stage. In many instances, the irregular heartbeats known as ectopic heartbeats appear intermittently without showing any serious symptoms. Such ectopic heartbeats are becoming quite common among healthy population and are often getting unnoticed.

Nowadays, several clinical practices are used to monitor the cardiac abnormalities such as ECG, magnetic resonance imaging (MRI), computerized tomography scan (CT scan), Echocardiography (Echo), Nuclear myocardial perfusion scan etc., [6]. Among the mentioned clinical practices, ECG is a well-established and widely adopted practice to monitor physiological activities of the heart. However, ECG provides shallow information on the functioning of various cardiac events. Moreover, a study conducted in [7] recommends that sole use of ECG is not reliable for the diagnosis of serious cardiac abnormalities. On the other hand, MRI, CT scan, and Echo are reliable practices over ECG, but they are highly expensive, time-consuming, and labor-intensive, which often require expertise to carry out [6].

Different from the aforementioned clinical practices, non-invasive acquisition of cardiac signals such as seismocardiogram (SCG) and ballistocardiogram (BCG) are also potential low-cost alternatives to monitor cardiac mechanical activities. SCG is an accelerometer sensor based modality that can record ultra-low-frequency vibrations of cardiac cycle mechanics along with timings of corresponding cardiac events [8]. Although BCG and SCG can record cardiac mechanical activities, diagnosis based on them is still in the premature stage to be considered for clinical practices. However, recent literature [6,8,9,10,11] show growing confidence on the applicability of SCG in clinical practices over BCG. Moreover, SCG is a noninvasive as well as an inexpensive accelerometer-based cardiac recording method, which can easily be carried out using wearable sensors. Hence, in this proposal, SCG is explored as an additional measure along with the ECG to design a robust cardiac health monitoring system.

In past, it was difficult to gather a huge amount of continuous cardiac data such as ECG and SCG to carry out comprehensive analysis. However, recent advancements in wearable technology have made it possible to collect cardiac data in an easy and affordable manner via wearable sensors for the longer duration. Although most cardiac abnormalities often appear only intermittently, they need to be registered, tracked and analyzed thoroughly as time goes by. This encourages us to design an early warning system, where continuously generated cardiac data are analyzed thoroughly to capture early signs of cardiac abnormal behavior.

The rest of the paper is organized as follows. Section 2 describes the related works followed by motivation and goals of the paper. The system model is presented in Section 3. Cardiological data analysis is described in Section 4. Performance evaluation is carried out in Section 5. Results and discussions are made in Section 6. Concluding remarks and future works are discussed in Section 7.

## 2. Related Works

In the past, several efforts are made to remotely monitor various physiological parameters of a user using affordable body sensors in a continuous manner. The *LifeGuard* [12] is one of the early efforts that monitors electrocardiogram, heart rate, respiration rate, temperature and blood pressure to alert the users. The *LifeGuard* equipped system transfers the health parameters to the base station via Bluetooth-enabled cell phone and raises the buzzer alarm, whenever abnormalities are observed. Similarly, in [13], ECG based body networks are proposed that raise an alarm if a heart attack is detected. However, many times due to bad signal quality or intense physical activities by a user, systems may raise false alarms under normal circumstances as well. In [14], a false arrhythmia alarm reduction framework is proposed using machine learning. In [15], ECG-based automatic recognition of arrhythmias is proposed for the diagnosis of heart diseases. Usually, remote monitoring of a person becomes a challenging job, if a person is engaged in activities such as motor racing, cycling, car racing and on field military service. The *Smart Helmet* is specifically designed to address the aforementioned challenges and proposes a bio-sensors equipped embedded helmet to monitor ECG and respiration of a user in an uninterrupted manner [16].

The another recent effort to remotely monitor cardiovascular and respiratory variables is a mobile healthcare platform *PlaIMoS* [17]. The conceived *PlaIMoS* architectural platform typically deals with data collection, communication, analysis and visualization of various healthcare settings. In continuously generated large data sets of ECG patterns, it is highly challenging to visualize cardiac health information aggregated over the period of time. Under such circumstances, easy-to-interpret visualization facility on cardiac health information is highly desirable to distinguish between healthy and abnormal cardiac settings. The *ECG Clock Generator* [18] is an attempt to provide visualization facility of cardiac activities accumulated over the period of time using ECG patterns.

In addition to ECG, in recent times, seismocardiogram (SCG) signals are also used to monitor the vital cardiac health parameters. In [19], authors have designed seismocardiography using tri-axial accelerometer embedded with electrocardiogram. Recently, Di Rienzo et al. [20] have designed accelerometer sensors based smart garment to record as well as monitor ECG, SCG, and respiratory variables of an ambulant subject out-of-laboratory setting. SCG also has potential clinical applications such as real-time heart rate monitoring [21] and left ventricular health monitoring [22] from wearable SCG measurements.

The raw SCG signals are less informative unless specific peaks are identified and correlated with underlying cardiac activities. A typical SCG cardiac cycle includes a set of nine relevant peaks, also called as feature points such as atrial systole AS, closing of mitral valve MC, opening of aortic valve AO, rapid systolic ejection RE, closing of aortic valve AC, opening of mitral valve MO, rapid diastolic filling RF, isovolumic movement IM, and isovolumic contraction IC. Various hemodynamic parameters can be estimated using SCG feature points. For example, in [23] systolic time intervals such as pre-ejection time (PEP), left ventricle ejection time (LVET), and electromechanical systole (QS2) are estimated by identifying SCG feature points AO, AC, IM, MO, and MC. Similarly, in [8], cardiac time intervals PEP, LVET, systolic time (SYS), and diastolic time (DIA) are estimated by identifying relevant SCG feature points.

In recent years, SCG shows numerous applications in real-time continuous health monitoring. For example, SCG is employed for the detection of respiratory phases in [9] such as inhale or exhale, which subsequently enable the estimation of systolic time intervals. In [11], the heart condition is estimated from the learned morphology of seismocardiography. In [24], SCG based automated detection of atrial fibrillation is demonstrated. In [25], a comparative study on pulse transit time measurement using seismocardiography, photoplethysmography and acoustic recordings is carried out. In [10], a cardiac early warning system using wearable ECG and SCG signals is introduced. In [6], a multichannel SCG is employed and six location-specific feature points are identified in addition to nine SCG feature points described previously.

To the best of our knowledge, the state-of-art recent studies [6,8,23] exclusively focus on separate or combined annotation of ECG and SCG or focus on the annotation based disease-specific cardiac function monitoring [9,24]. However, mere annotation of morphologies may not provide the powerful insight unless the comprehensive theoretical analysis is explored. Since ECG and SCG are pseudo-accurate in nature, their exclusive usage for the cardiac health monitoring limits the scope of the improvement. It is known that ECG signals represent only cardiac electrical activities and all heart problems cannot be detected by analyzing these ECG signals. For example, a common heart disorder known as angina may not usually appear in routine ECG. In addition, vulnerable plaque deposition, which is mainly responsible for asymptomatic blockages in heart arteries, is difficult to capture by ECG and requires detailed investigation and tests. The Seismocardiogram (SCG) can be employed to complement the ECG as SCG represents the cardiac mechanical activities and provides in-depth meaningful insights of the mechanical functioning of the heart. However, SCG has also few limitations such as lack of well studied features to determine the heart conditions such as hypertensive heart disease. Hence, feature points based beat-by-beat combined analysis of ECG and SCG is expected to bring additional information to carry out the detail analysis for continuous cardiac health monitoring. It is likely that joint analysis of ECG and SCG can complement to each other to produce more reliable outcomes. The combined analysis of ECG and SCG is beneficial as the outcome of each heartbeat is validated based on the combined knowledge of the cardiac electrical and mechanical activities.

### Motivation and Goals

It is increasingly becoming important to record, monitor and investigate the intermittent cardiac anomalies those appear due to the unhealthy lifestyle of people leading to CVD. Usually, cardiac anomalies may occur only intermittently and they go completely unnoticed leading to a sudden death of a cardiac patient. Moreover, ECG signals of cardiac patients also stay entirely normal on regular basis except during those intermittent cardiac anomalies. Under such circumstances, it is highly challenging to foresee potential cardiac health problems in the early stage using traditional in-hospital ECG based diagnosis.

To capture intermittent cardiac anomalies, various cardiac health parameters need to be recorded and monitored for the longer duration of time, which can be accomplished via wearable ECG and SCG sensors. However, raw ECG and SCG signals are less informative unless signals are properly annotated and thoroughly investigated beat by beat. The manual annotation of feature points is a tedious, cumbersome and time-consuming process. In addition, the manual annotation is not a viable approach to design a continuous cardiac health monitoring system. Hence, the computer-assisted automatic annotation of ECG and SCG is highly essential. Additionally, feature points based cardiac health monitoring methods are needed to distinguish the morphology of any cardiac cycle between normal and abnormal. In the proposed study, by use of phrase *anomalies*, we mean the unusual morphology of ECG and SCG in terms of uncommon recording of an amplitude of peaks, and duration of waves, segments, and intervals. It is to be noted that the mere presence of uncommon recordings in a single cardiac cycle does not mean the cardiac anomalies. For this reason, the set of cardiac cycles is needed to be monitored to help capture developing trends for the cardiac early warnings.

Since cardiac health monitoring is a very broad area, we limit the scope of our investigation only to jointly monitor the normal vs. abnormal behavior of cardiac cycles in simultaneous ECG and SCG recordings. The main objective is to present the viability of joint investigation of ECG and SCG for long-term cardiac health monitoring. In the future, the study can be extended to accommodate the urgency of the abnormality. The goals of our study are listed as follows:

1. **Design a body sensor network to collect the simultaneous ECG and SCG signals;**
2. **Design automatic feature point delineation algorithm for annotation of ECG and SCG signals;**
3. **Design abnormal morphology detection method for ECG signals;**
4. **Design abnormal morphology detection method for SCG signals;**
5. **Design combined analysis model for ECG and SCG signals.**

## 3. System Model

**Figure 1:**
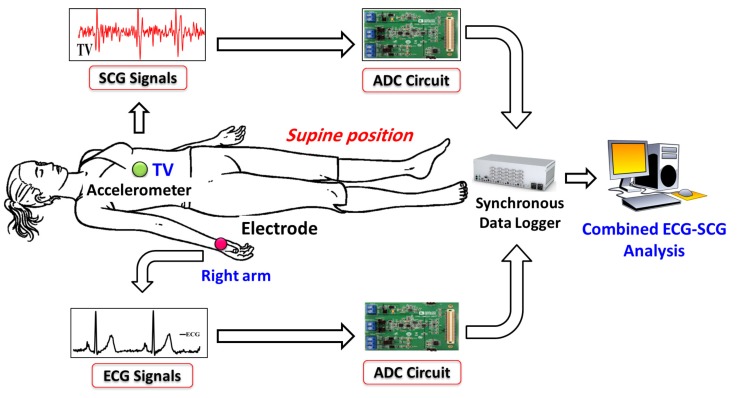
Architectural view of ECG/SCG data collection model.

The architectural view of the proposed ECG and SCG data collection model is shown in Figure 1. For SCG data collection, an SCG sensing module is placed at a valvular auscultation site called a Tricuspid valve TV, and ECG data collection is carried out placing an ECG sensing module (e.g., electrode) at the right arm as shown in Figure 1. The high quality disposable electrode H135SG Covidien from Bio-Medical Instruments (Clinton Township, MI, USA) [26] is used as an ECG sensing module. The accelerometer sensor LIS331DLH from STMicroelectronics (Geneva, Switzerland) [27] is used as a core component of SCG sensing module. The sensing ability, sensing range and gravitational force sensitivity of SCG sensing module is set to 0.5 Hz to 1 kHz, +2 g to –2 g, and 1 mg, respectively. The band pass filter with frequency 0.5 Hz–50 Hz is applied analogically to get the required ECG and SCG signals at sampling frequency of 1000 Hz. The microcontroller system ADuC7020 from Analog Devices Inc. (Cambridge, MA, USA) [28] is used for the communication from ECG/SCG sensing modules to Analog-to-Digital convert (ADC) circuit and the PowerLab 16/35 from AD Instruments (Dunedin, New Zealand) [29] is used as the synchronous data logger, which further amplifies and filters the concurrent signals. The class of nonlinear filters also known as filter bank presented in [30] is employed for the noise reduction and baseline wander removal with minimal signal distortion. It is reported that the nonlinear filters are expected to perform better than other baseline wander removal methods such as adaptive filters, moving average filters, etc. [30].

The SCG signals acquired from Tricuspid valve and lead *I* ECG signals are used for the combined analysis. The conventional Tricuspid valve site is chosen as the interventricular septum is located beneath the Tricuspid valve, which provides more clear signals. During the entire data collection process, the heart rate is monitored using Finger-clip sensor PAH8001EI-2G [31] and the respiratory rates are monitored manually to ensure the stability and resting position of the subjects. The data collection is performed in three sessions per subject with at least 5 min of break between the sessions. The described data collection procedure is comprehensively verified and approved by Institutional Review Board (IRB) of the Chang Gung Memorial Hospital (CGMH), Taoyuan, Taiwan with IRB license number 104-6615B.

## 4. Cardiological Data Analysis

**Figure 2:**
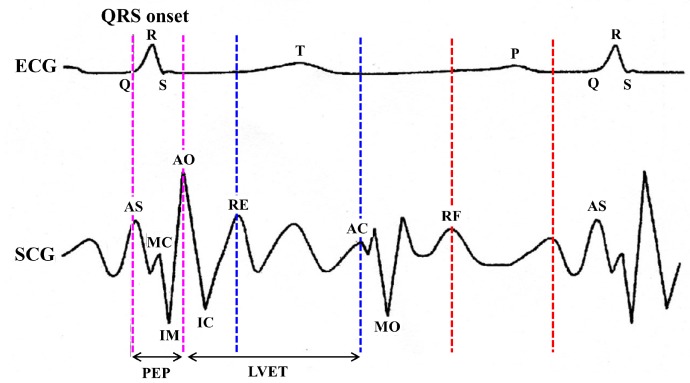
Cardiac electrical and mechanical activities.

In this section, we introduce various feature points of ECG and SCG along with corresponding values to distinguish between normal and abnormal cardiological data, followed by ECG/SCG based feature points delineations and cardiac health monitoring methods. The ECG and SCG cardiac signals with corresponding cardiac electrical and mechanical activities are explained as shown in Figure 2 using the normal ECG waveform aligned with the normal SCG waveform. The ventricle depolarization, a cardiac electrical activity that takes place during the QRS complex can be represented by the corresponding cardiac mechanical activities that take place between atrial systole AS to the opening of aortic valve AO. Similarly, during the ventricle re-polarization (*T* wave) of ECG, the cardiac mechanical activity such as rapid ejection of blood flow RE takes place until the closing of aortic valve AC. Finally, during the atrial depolarization represented as *P* wave, cardiac mechanical activity known as rapid diastolic filling RF can be observed.

### 4.1. Differentiation between Normal and Abnormal ECG Morphology

**Figure 3:**
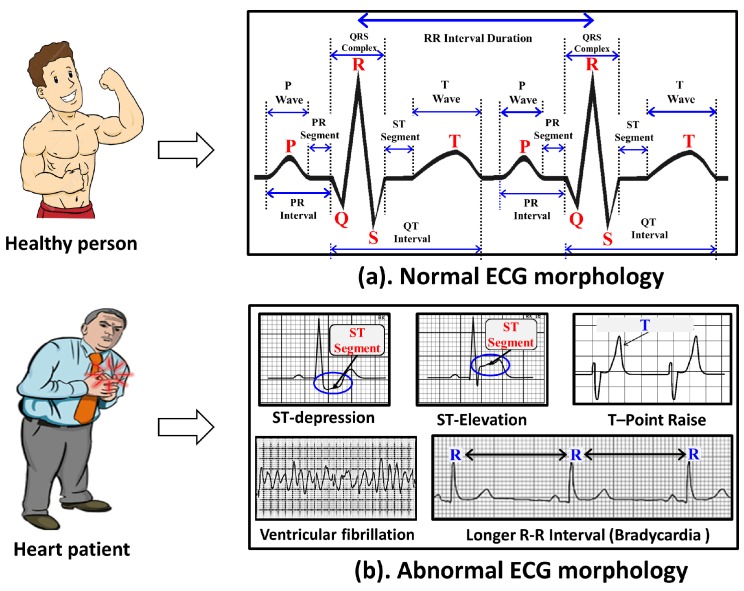
Example of normal and abnormal ECG morphologies.

For a normal and healthy heart, each heartbeat reflects an orderly progression of depolarization in ECG tracing, which is helpful to know various heart functionalities. As shown in Figure 3a, the normal ECG cycle is comprised of several cardiac electrical activities known as depolarization and re-polarization responsible for heart muscular activities. The entire process of depolarization and re-polarization of a cardiac cycle can be explained as follows. The ECG *P* wave represents the atrial depolarization spreads from sinoatrial (SA) node throughout the atria followed by brief period of zero voltage isoelectric representing delay at atrioventricular (AV) node. The QRS complex represents the short duration of ventricular depolarization followed by ventricular re-polarization represented by *T* wave. The ST segment between QRS complex and *T* wave represents the brief period of zero voltage isoelectric, when both ventricles are completely depolarized. To define the normal and abnormal behavior of depolarization, ECG trace is first divided into a set of heartbeat cycles. Each heartbeat cycle is again sub-divided into various waves, segments, and interval such as *P* wave, QRS wave, *T* wave, PR segment, ST segment, PR interval, and QT interval. The subdivision of heart beat cycle into waves, segments, and intervals is performed based on the position and order of the ECG cardiac feature points *P*, *Q*, *R*, *S* and *T* as shown in Figure 3a. In addition, RR interval duration can also be used as a measure to decide between the normal and abnormal behavior of two consecutive heartbeats.

Table 1 shows the notations used in this paper to represent reference maximum and a minimum value of waves, segments, and intervals observed in a normal ECG tracing. Cardiac anomalies such as myocardial infarction, ischemia, sinus arrhythmia, sinus bradycardia, atrial/ventricular fibrillation, etc., disturb the orderly progression of depolarization, and hence morphology of various waves, segments and intervals changes significantly. Figure 3b shows various prominent ECG anomalies. For example, in ST depression, a line at ST segment significantly bends downward below the isoelectric line due to stable/unstable angina problem of a patient. On the other hand, in ST elevation, a line at ST segment bends significantly upward above the isoelectric line due to non-transmural ischemia. Moreover, bradycardia, characterized by a longer RR interval, can cause symptoms such as dizziness, fatigue, chest pain etc. Although ECG is widely used to identify various cardiac anomalies, it may lead to the wrong diagnosis and may falsely indicate the presence of CVD in patients with minor symptoms of negligible risk to CVD [7]. Hence, it is necessary to correlate the abnormal behaviors observed in ECG trace with the corresponding SCG trace to ensure the reliable cardiac health monitoring. The following section describes the process of feature points delineation for ECG and SCG traces.

### 4.2. Feature Points Delineation Mechanism

**Figure 4:**
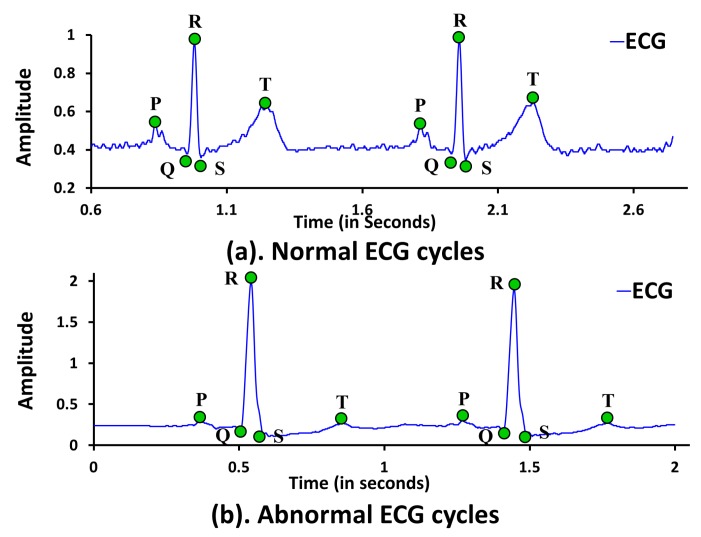
Feature points delineation in (**a**) normal ECG cycles; (**b**) abnormal ECG cycles.

**Figure 5:**
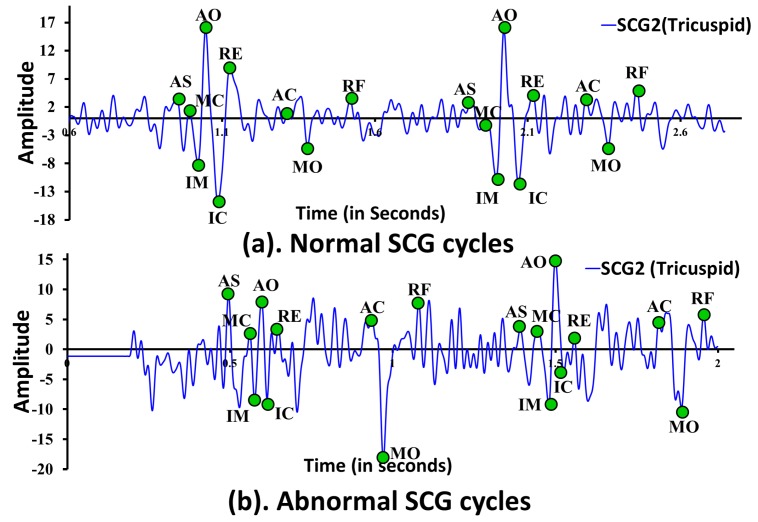
Feature points delineation in (**a**) normal SCG cycles; (**b**) abnormal SCG cycles.

In this subsection, we present two separate mechanisms, one for ECG and another for SCG, to select the corresponding feature points. Five ECG feature points such as *P*, *Q*, *R*, *S*, and *T*, and nine SCG feature points such as AS, MC, IM, AO, IC, RE, AC, MO, and RF are considered. Figure 4 shows an example normal/abnormal ECG trace along with five ECG feature points selected by using the proposed ECG feature points delineation algorithm. Similarly, Figure 5 shows an example SCG trace with nine SCG feature points selected by the proposed SCG feature point delineation mechanism. However, it is expected that the proposed ECG/SCG feature point delineation mechanisms should select the corresponding feature points from normal as well as abnormal ECG/SCG traces, it is to note that cardiac anomalies such as ventricular fibrillation shown in Figure 3b may result in non-detection of few or all feature points. Here, it is assumed that ECG and SCG traces are collected in the form of vectors of data points represented as $V_{ecg}$ and $V_{scg}$ with known sampling rate $S_{r}$ and mean heart rate $H_{r}$. Each sampled ECG and SCG data point in $V_{ecg}$ and $V_{scg}$ represents unique amplitude value in terms of millivolts (mV). All of the local maximum and minimum peaks in ECG/SCG cycles are identified using second derivatives with slope value zero to measure the corresponding ECG/SCG amplitude. The measured amplitude represents the sensor value minus the baseline, where the baseline amplitude is computed as zero voltage of ECG/SCG signal. For feature points’ delineation, both methodologies first select the feature points in the first cardiac cycle and continue to select the feature points in subsequent cardiac cycles with a minimum separation distance equivalent to the cardiac cycle length CL between the same feature points. The sampling rate $S_{r}$ and mean heart rate $H_{r}$ are the known input parameters of the experimental data sets used to estimate the cardiac cycle length represented as $CL=\frac{1}{H_{r}}*S_{r}$. In practice, $H_{r}$ is estimated continuously from the RR interval duration and updated to continuously estimate the CL.

#### 4.2.1. ECG Feature Points Delineation Mechanism

**Figure 6:**
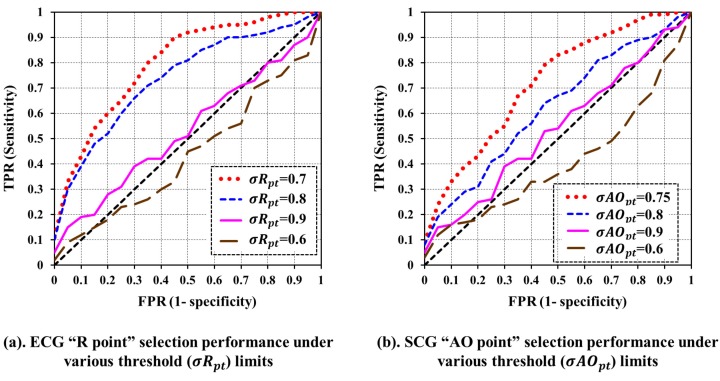
Evaluation of delineation of ECG feature point *R* and SCG feature point AO.

For ECG trace, at first, the feature point *R* is selected and then after rest feature points *Q*, *R*, *S* and *T* are selected with respect to *R* considering the referenced normal values of various waves, intervals, and segments as reported in [32,33]. Although normal referenced values may not be the best indicators to detect the very specific heart diseases, they can act as sufficient estimators under quite a bit distorted morphologies for the unobtrusive cardiac health monitoring. Normally, feature point *R* exhibits high amplitude, which is easy to detect. The process of feature point *R* detection can be described as follows. Firstly, a unique peak $\zeta_{pt}$ exhibiting the maximum amplitude is identified from all the cardiac cycles under consideration. Later, all the peaks in a cardiac cycle with amplitude greater than $\sigma R_{pt}\times \zeta_{pt}$ are chosen as candidate *R* peaks represented as $cnR_{pts}$. Here, $\sigma R_{pt}$ is a constant between 0 and 1, which must be determined experimentally. In the current study, the $\sigma R_{pt}=0.7$ is obtained experimentally, which provides consistently superior performance as shown in Figure 6a. Finally, the peak exhibiting maximum amplitude among $cnR_{pts}$ in a cardiac cycle is designated as feature point *R* and is represented by $R_{pt}$.

The feature points’ delineation other than *R* is a non-trivial process, and therefore feature point specific range is formulated in such a way that it maximizes the chances of respective feature point delineation. Four ranges of data points represented as $Q_{rg}$, $P_{rg}$, $S_{rg}$, and $T_{rg}$ are formulated with respect to the feature point *R* to select ECG feature points *Q*, *P*, *S* and *T*, respectively. The range format for feature points appearing before and after *R* is formulated as $\left(R_{pt}-Y,R_{pt}\right)$ and $\left(R_{pt},R_{pt}+\left(Y+\alpha \right)\right)$, respectively. Here, *Y* represents the set of data points equivalent to the normal wave duration of the corresponding feature point. For example, for feature point *Q*, $Y=\frac{\Delta QRS_{wv}*S_{r}}{2}$. However, to reduce the estimation error of *Y*, an error margin constant α is added as a precautionary measure to get better estimation of feature-point range *Y*. Here, $\Delta QRS_{wv}$ represents the normal time duration of the QRS wave, and $S_{r}$ represents the sampling frequency. For each feature point, the corresponding peak is identified and designated as a feature point based on the minima and maxima characteristic. For example, the peak representing the feature point *Q* in ECG normally appears as minima and therefore the minimum peak from the range is designated as $Q_{pt}$. A similar process is repeated for each feature point except *R* in each ECG cardiac cycle. The entire process in the form of pseudo-code is given in Algorithm 1.

**Figure 7:**
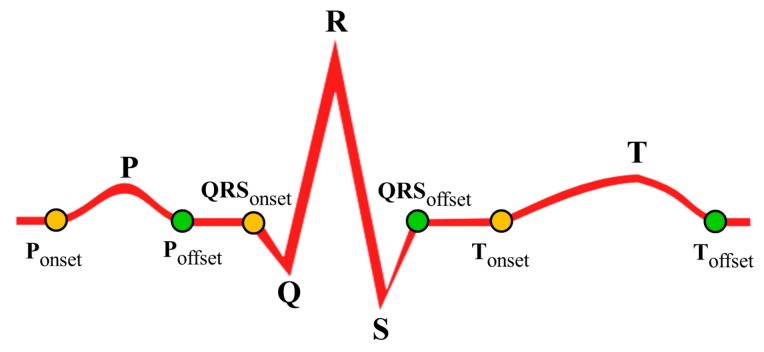
Example of ECG feature points, onset points and offset points.

Besides delineation of ECG feature points, it is also essential to delineate end points of the waves. The ECG end points of waves can be classified into two sets. The set of onset points such as $\{P_{onset}$, $QRS_{onset}$, $T_{onset}\}$, and the set of offset points $\{P_{offset}$, $QRS_{offset}$, $T_{offset}\}$ as shown in Figure 7. In many instances, due to the cardiac abnormalities such as left/right atrial enlargement, ST elevation/depression, and *T* point raise, the delineation of end points become difficult. In particular, the delineation of end points in case of flatten/inverted *T* waves is painful. Hence, a simple end point delineation mechanism is incorporated. At first, using the duration of *P* wave, QRS complex and *T* wave observed in normal ECG cycles, the set of onset range $\{P_{onset}^{rg}$, $QRS_{onset}^{rg}$, $T_{onset}^{rg}\}$ and the set of offset range $\{P_{offset}^{rg}$, $QRS_{offset}^{rg}$, $T_{offset}^{rg}\}$ are derived with respect to the feature points *P*, *R* and *T*, respectively. Finally, the data point with minimum amplitude value is located within the range $P_{onset}^{rg}$, $T_{onset}^{rg}$, $P_{offset}^{rg}$ and $T_{offset}^{rg}$ and annotated with $P_{onset}$, $T_{onset}$, $P_{offset}$ and $T_{offset}$, respectively. Similarly, $QRS_{onset}$ and $QRS_{offset}$ are located as the maximum data points within the range $QRS_{onset}^{rg}$ and $QRS_{offset}^{rg}$, respectively.

#### 4.2.2. SCG Feature Points Delineation Mechanism

Similar approach of ECG is adopted while selecting various feature points from the SCG trace, where, at first, feature point AO is selected and then rest eight feature points AS, MC, IM, IC, RE, AC, MO, and RF are selected with respect to the position of AO. One of the distinguished properties of SCG is the unusual high amplitude of feature point AO in SCG morphology as shown in Figure 5, which makes it easy for the delineation of AO in a cardiac cycle. However, this does not hold true in every cardiac cycles and is valid under the specific constraints. For example, in a clear SCG morphology of a healthy subject, AO normally exhibits high amplitude in most cycles with few exceptions. However, in distorted SCG morphology of an unhealthy subject, the delineation of AO is more complicated and may give poor results. Similar to selecting *R* in ECG feature point delineation, maximum amplitude peak $\zeta_{pt}$ from the set of cardiac cycles is located first. Then, for each individual cardiac cycle, the set of candidate AO peaks represented as $cnAO_{pts}$ are located with amplitude more than $\sigma AO_{pt}\times \zeta_{pt}$. Here, $\sigma AO_{pt}$ is a user-defined constant between 0 and 1, which can be obtained experimentally. In the current study, $\sigma AO_{pt}=0.75$ is found to give consistently superior performance as shown in Figure 6b. Finally, from the set $cnAO_{pts}$, a unique peak exhibiting maximum amplitude is designated as SCG feature point AO represented by $AO_{pt}$.

SCG morphology is more complex in nature as compared to ECG and therefore it requires significant efforts to correctly locate the feature points other than AO. Since SCG morphology is not well studied, the normal representative value of an amplitude of various peaks and duration of waves are not yet well defined in the literature. Hence, set of training SCG cardiac cycles from the healthy subjects are used to estimate the distance (i.e., the window size) of various SCG feature points with respect to AO to formulate feature point specific range. The window size calculated from the training SCG cycles of healthy population has dual advantages: (1) it helps to estimate the normal window size for healthy subjects; and (2) it also helps to identify the significantly varying abnormal SCG features lying outside the normal window size (e.g., outliers), which is observed among non-healthy subjects. For each SCG feature point, a fixed size range $X_{rg}$ consisting of probable data points is formulated at the obtained feature point specific window size SW(X), where X∈{AS,MC,IM,IC,RE,AC,MO,RF}. Finally, based on the morphological maxima or minima characteristic of each feature point, a unique peak is obtained from the $X_{rg}$ and is designated as a feature point. For example, for SCG feature point AS, range $AS_{rg}$ of probable data points for AS at distance SW(AS) is formulated with respect to AO. Subsequently, the peak with maximum amplitude in the range of $AS_{rg}+\alpha$ is designated as SCG feature point $AS_{pt}$. A similar approach is followed for the delineation of all other SCG feature points. Detailed pseudo code for SCG feature points’ delineation is described in Algorithm 2.

**Algorithm 1:** Delineation of ECG feature points. **Input**:  $V_{ecg}:$ Vector of ECG data points with amplitude, $S_{r}:$ ECG data sampling rate, $H_{r}:$ Heart rate, RFV: Set of referenced feature values. **Output**:  $F_{ecg}=\left\{P,Q,R,S,T\right\}:$ ECG feature point set. **Notations**:  $\zeta_{pt}$: Maximum amplitude data point,  $cnR_{pts}$: Candidate *R* points,  $X_{rg}$: Range of points to locate point *X*, where X∈{P,Q,S,T},  α: User defined error margin constant,  $\sigma R_{pt}\in \left(0,1\right)$: Constant to detect *R* points,  $F_{ecg}^{a}$: ECG feature point set for $a^{th}$cardiac cycle. **1** Initialize $F_{ecg}=null$; **2** Estimate cardiac cycle length: $CL=\frac{S_{r}}{H_{r}}$; **3** Calculate # of cardiac cycles: $CCs=\frac{\left|V_{ecg}\right|}{CL}$; **4** Select maximum amplitude data point: $\zeta_{pt}$
$=max(V_{ecg}^{1},\ldots ,V_{ecg}^{\left|V_{ecg}\right|})$; **5**
**for**
a=1
**to**
CCs
**do** **6**  Initialize $F_{ecg}^{a}=null$; **7**  Select candidate *R* points: $cnR_{pts}=\left(V_{ecg}^{j}\geq \sigma R_{pt}*\zeta_{pt}\right)$, $\forall j\in V_{ecg}$; **8**  $R_{pt}=max\left(cnR_{pts}\right)$; // Delineation of feature point *R*.; **9**  $F_{ecg}^{a}=F_{ecg}^{a}\cup R_{pt}$; **10**  $Q_{rg}=\left(R_{pt}-\left(\frac{\Delta QRS_{wv}*S_{r}}{2}+\alpha \right),R_{pt}\right)$; **11**  $Q_{pt}=min\left(V_{ecg}^{j}\right)$, $\forall j\in Q_{rg}$; // Delineation of feature point *Q*.; **12**  $F_{ecg}^{a}=F_{ecg}^{a}\cup Q_{pt}$; **13**  $P_{rg}=\left(R_{pt}-\left(\Delta PR_{inv}*S_{r}+\alpha \right),R_{pt}\right)$; **14**  $P_{pt}=max\left(V_{ecg}^{j}\right)$, $\forall j\in P_{rg}$; // Delineation of feature point *P*.; **15**  $F_{ecg}^{a}=F_{ecg}^{a}\cup P_{pt}$; **16**  $S_{rg}=\left(R_{pt},R_{pt}+\left(\frac{\Delta QRS_{wv}*S_{r}}{2}+\alpha \right)\right)$; **17**  $S_{pt}=min\left(V_{ecg}^{j}\right)$, $\forall j\in S_{rg}$; // Delineation of feature point *S*.; **18**  $F_{ecg}^{a}=F_{ecg}^{a}\cup QRS_{pt}$; **19**  $T_{rg}=\left(R_{pt},R_{pt}+\left(\Delta QT_{inv}*S_{r}-\frac{\Delta QRS_{wv}*S_{r}}{2}+\alpha \right)\right)$; **20**  $T_{pt}=max\left(V_{ecg}^{j}\right)$, $\forall j\in T_{rg}$; // Delineation of feature point *T*.; **21**  $F_{ecg}^{a}=F_{ecg}^{a}\cup T_{pt}$; **22**  $F_{ecg}=F_{ecg}\cup F_{ecg}^{a}$; **23**
**endfor** **24** return $F_{ecg}$;

### 4.3. Data Analysis Methodology

In this subsection, we present mechanisms to differentiate between normal and abnormal morphology of ECG and SCG cardiac cycles.

#### 4.3.1. Abnormal ECG Morphology Detection

The cardiac abnormal morphology in ECG trace either appear in the form of significant variation in amplitude of feature points *P*, *Q*, *R*, *S*, and *T*, or appear in the form of significant variation in the duration of waves i.e., *P*, QRS, *T*, segments i.e., ST, PR, and intervals i.e., RR, PR, ST, and QT. In this paper, the abnormal morphology related to *P* wave, QRS complex, and *T* wave is determined with respect to the referenced amplitude and wave durations as suggested in [32]. It is observed that critical cardiac anomalies in a patient normally last a little longer and affect multiple consecutive heartbeats. Therefore, cardiac abnormalities that arise in an individual heartbeat due to occasional uncommon amplitude and duration are ignored. Instead of monitoring cardiac cycles individually, the group of cardiac cycles is monitored together such as 5-cycles, 10-cycles, 25-cycles, and 50-cycles, etc. The purpose of considering the group of cardiac cycles is to ensure that robust cardiac abnormalities are captured by the system instead of the system being misguided by occasional false abnormalities that arise due to bad signal quality and external noises.

The cardiac abnormal morphology count is maintained, which is increased each time by one, whenever amplitude, wave duration, or both vary substantially with respect to their corresponding reference values. Finally, at the completion of the group of cardiac cycles, a feature point with a maximum number of cardiac abnormalities is considered as the abnormal feature point. The pseudo-code for abnormal morphology detection in ECG trace for the group of *k*-cycles is shown in Algorithm 3, where *k* is a user-defined constant. Here, it is assumed that ECG signals are available in the form of vector $V_{ecg}^{i}$, where $i=\{1,2,\ldots ,\left|V_{ecg}\right|\}$ represents set of data points sampled with sampling rate $S_{r}$.

**Algorithm 2:** Delineation of SCG feature points.
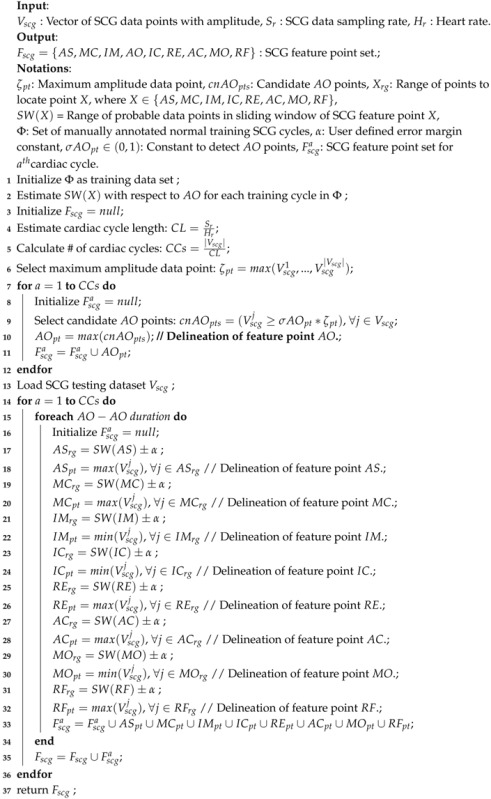


**Algorithm 3:** Abnormal ECG morphology detection **Input**:  $V_{ecg}:$ Vector of ECG data points with amplitude, $S_{r}:$ ECG data sampling rate,  $H_{r}:$ Heart rate, RFV: Set of referenced feature values, k: User defined constant to group *k*-cycles. **Output**: Detection of abnormal morphologies **Notations**:  $\theta X_{wv}$: Measured amplitude of *X* wave,  $\psi X_{wv}$: Measured duration of *X* wave,  $cX_{wv}$: Counter for *X* wave abnormal morphologies, where X∈{P,QRS,T},  $cRR_{inv}$: Counter for RR interval abnormal morphologies,  $\psi RR_{inv}$: Measured duration of RR interval. **1** Initialize *k*; **2** Initialize $cP_{wv}=cQRS_{wv}=cT_{wv}=cRR_{inv}=0$; **3** Estimate Cardiac cycle Length: $CL=\frac{S_{r}}{H_{r}}$; **4** Calculate # of cardiac cycles: $CCs=\frac{\left|V_{ecg}\right|}{CL}$; **5**
**for**
*i=1*
**to**
*CCs−k+1*
**do** **6**  j=i; **7**  **while**
*j≤(i+k)*
**do** **8**  **if**
$\left(\theta P_{wv}>\Omega P_{wv}\right)\vee \left(\theta P_{wv}<\omega P_{wv}\right)\vee \left(\psi P_{wv}>\Delta P_{wv}\right)\vee \left(\psi P_{wv}<\delta P_{wv}\right)$
**then** **9**   $cP_{wv}=cP_{wv}+1$; // *P* wave abnormal morphology detection **10**  **end** **11**  ; **12**  **if**
$\left(\theta QRS_{wv}>\Omega QRS_{wv}\right)\vee \left(\theta QRS_{wv}<\omega QRS_{wv}\right)\vee \left(\psi QRS_{wv}>\Delta QRS_{wv}\right)\vee \left(\psi QRS_{wv}<\delta P_{wv}\right)$
**then** **13**   $cQRS_{wv}=cQRS_{wv}+1$; // QRS wave abnormal morphology detection; **14**  **end** **15**  **if**
$\left(\theta T_{wv}>\Omega T_{wv}\right)\vee \left(\theta T_{wv}<\omega T_{wv}\right)\vee \left(\psi T_{wv}>\Delta T_{wv}\right)\vee \left(\psi T_{wv}<\delta T_{wv}\right)$
**then** **16**   $cT_{wv}=cT_{wv}+1$; // *T* wave abnormal morphology detection; **17**  **end** **18**  **if**
$\left(\psi RR_{inv}>\Delta RR_{inv}\right)\vee \left(\psi RR_{inv}<\delta RR_{inv}\right)$
**then** **19**   $cRR_{inv}=cRR_{inv}+1$; // $RR_{inv}$ interval abnormal morphology detection;
**20**   **end** **21**  **end** **22**
**endfor**

At first, from the past studies [32,33], a set of reference values RFV is formulated. The RFV consists of reference normal values corresponding to an amplitude of feature points and duration of waves, segments, and intervals. The set RFV represents the reference normal values within two standard deviations from the mean with percentile range of 2% through 98% of healthy subjects. Table 1 defines the notations that are used to represent the reference values used in Algorithm 3. $V_{ecg}^{i}$, $S_{r}$, $H_{r}$, and a set RFV are considered as input to Algorithm 3 to detect the cardiac abnormal cycles in ECG trace. In each cardiac cycle, the current measured value of amplitude, wave, segment, and interval duration of each feature point is compared with the corresponding reference normal values. As shown in Algorithm 3, in cardiac abnormality detection of *P*, the current measured value of amplitude $\theta P_{wv}$ and wave duration $\psi P_{wv}$ are compared with the reference minimum and maximum value of amplitude (i.e., $\Omega P_{wv}$ and $\omega P_{wv}$) and wave duration (i.e., $\Delta P_{wv}$ and $\delta P_{wv}$), respectively. The *P* wave cardiac abnormal morphology counter $cP_{wv}$ is maintained, which is increased by one in each time, either measured amplitude $\theta P_{wv}$ or wave duration $\psi P_{wv}$ lies outside the reference normal values. The similar approach is followed to detect the abnormal morphology of $QRS_{wv}$, $T_{wv}$ and $RR_{inv}$.

#### 4.3.2. Abnormal SCG Morphology Detection

**Figure 8:**
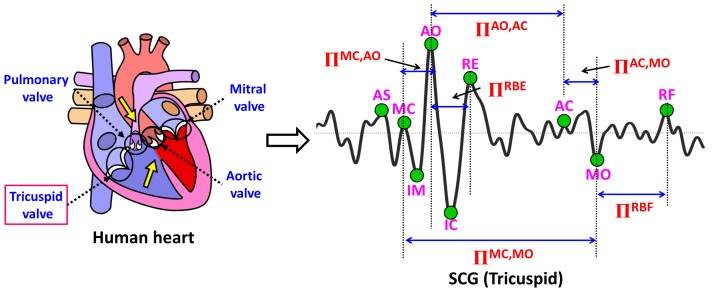
Feature-variables derived from SCG Tricuspid valve site.

As far as our knowledge is concerned, there exists no well-studied method to detect the abnormal morphology of SCG cardiac cycles. The existing literature mainly focuses on the delineation of SCG feature points [6,8,23] or focuses on the applicability of SCG in the diagnosis of cardiac health problems [20,23,24,25]. In this subsection, a process to identify abnormal morphologies in an SCG cardiac cycle is described. Since SCG is employed as an additional measure to complement the performance of ECG based monitoring, the SCG feature points’ delineation and designing of feature-variables are made independent from the ECG to avoid any sorts of performance influence on SCG. Six SCG feature variables are designed to detect morphological abnormalities in SCG cardiac cycles such as $\Pi^{MC,AO}$, $\Pi^{AO,AC}$, $\Pi^{MC,MO}$, $\Pi^{AC,MO}$, $\Pi^{RBE}$, and $\Pi^{RBF}$. These six SCG feature-variables represent the duration of various cardiac mechanical activities that take place in a cardiac cycle. The notation of SCG feature-variables along with their corresponding cardiac mechanical activities are described in Table 2. Due to the sensitive nature of SCG accelerometer sensors, the amplitude behavior of various SCG feature points is observed highly fluctuating. Therefore, the only parameter considered important to design SCG feature-variables is the duration of cardiac mechanical activities ignoring the amplitude parameter. In this paper, an SCG cardiac mechanical activity is considered abnormal, whenever significant variation is observed in the duration of corresponding SCG feature-variable with respect to the normal duration. Figure 8 shows the derivation of SCG feature-variables from the SCG signal.

In contrast to ECG, SCG does not have predefined referenced values to detect the abnormal behavior of feature-variables. Therefore, the reference value of each SCG feature-variable is first estimated from η number of initial SCG cardiac cycles from a set of five subjects. It is to be noted that the estimation of SCG reference values is the mean representation of five subjects. For a given subject and heart rate, these reference time intervals are first normalized and then the customized subject-specific set of reference time intervals is obtained. Later, the estimated value of feature-variables is used to measure the significant variation of feature-variables for a given subject. Here, η can be defined experimentally and it varies from one data set to another. The smaller the value of η, the more premature the estimation is observed and the higher the value of η, the more error propagation is observed. Hence, a trade-off needs to be balanced for optimum estimation of η. In this paper, the value of η is kept to 20 cardiac cycles, which consistently performs better to estimate the feature-variables with reasonable accuracy. The first η number of cardiac cycles is considered as an estimation phase, during which a cardiac abnormality detection process is not initiated; rather, duration of various feature-variables is estimated.

In the estimation phase, time series analysis of data is performed to smoothen out short-term fluctuations and to capture the long-term trend of feature-variables. Moreover, behavioral changes such as respirations and body movements are also accommodated in time-series signal analysis by assigning weights to the cardiac cycles in the decreasing order. The weighted moving average duration $WavgD_{i}$ and weighted moving standard deviation duration $WstdD_{i}$ are calculated for each individual feature-variable-*i*, where $i\in FV_{scg}$. At the end of the η number of cardiac cycles, the value of $WavgD_{i}$ and $WstdD_{i}$ are used as the decision values to detect the cardiac abnormalities in the duration of feature-variables in subsequent cardiac cycles.

In *estimation phase*, $WavgD_{i}^{k}$ is calculated using Equation (1) and $WstdD_{i}^{k}$ is calculated using Equations (2) and (3) for η number of cardiac cycles, where *i* represents the $i^{th}$ feature-variable and *k* represents the $k^{th}$ cardiac cycle. Here, $D_{i}^{k}$ represents the measured time duration of $i^{th}$ feature-variable in $k^{th}$ cardiac cycle, where $i\in FV_{scg}$:

(1) $$\begin{array}{c} WavgD_{i}^{k}=\left\{\begin{array}{cc} D_{i}^{k}, & if\;\;k<\eta ,\\ \frac{\sum_{k=1}^{\eta }k\times D_{i}^{k}}{\sum_{k=1}^{\eta }k}, & if\;\;k=\eta ,\\ WavgD_{i}^{k-1}+\frac{\eta }{\sum_{j=1}^{\eta }j}\left(D_{i}^{k}-WavgD_{i}^{k-1}\right), & if\;\;k>\eta . \end{array}\right. \end{array}$$

To calculate $WstdD_{i}^{k}$, continuous variance $S_{i}^{k}$ is first calculated using Equation (2). The method to calculate $S_{i}^{k}$ is inspired from B. P. Welford’s method [34], which is an accurate and guaranteed way to generate the non-negative variance under floating point calculations:

(2) $$\begin{array}{c} S_{i}^{k}=\left\{\begin{array}{cc} 0, & if\;k=1,\\ \frac{1}{\sum_{k=1}^{\eta }k}\sum_{k=1}^{\eta }\left(k\times {\left(D_{i}^{k}\right)}^{2}-WavgD_{i}^{k}\right), & if\;\;k=\eta ,\\ S_{i}^{k-1}\;+k\times \left(D_{i}^{k}-WavgD_{i}^{k-1}\right)*\left(D_{i}^{k}-WavgD_{i}^{k}\right), & if\;k>\eta . \end{array}\right. \end{array}$$

From variance $S_{i}^{k}$, weighted moving standard deviation duration $WstdD_{i}^{k}$ is calculated as shown in Equation (3):

(3) $$\begin{array}{cc} WstdD_{i}^{k}=\sqrt{\frac{S_{i}^{k}}{\left(k-1\right)}} & \;\;\;\;for\;\;k>\eta . \end{array}$$

Once the estimation phase is concluded and decision values $WavgD_{i}^{k}$ and $WstdD_{i}^{k}$ are obtained, *evaluation phase* is initiated. During each cycle in the *evaluation phase*, the value of each feature-variable $D_{i}^{k}$, where $i\in FV_{scg}$ and k>η is inspected against the range $\left(WavgD_{i}^{k}+WstdD_{i}^{k},WavgD_{i}^{k}-WstdD_{i}^{k}\right)$ of corresponding feature-variable. If value of any feature-variable $D_{i}^{k}$ is found lying outside the range $\left(WavgD_{i}^{k}+WstdD_{i}^{k},WavgD_{i}^{k}-WstdD_{i}^{k}\right)$, then the corresponding feature-variable is considered as potential outlier and it is marked as abnormal. Since the distribution of value of feature-variables with respect to the mean of corresponding feature-variable can be considered as Gaussian distribution, we used Chauvenet’s criterion [35] to identify outliers in each cycle of the evaluation phase. In order to identify the outliers, deviation $devD_{i}$ and tolerance $tolD_{i}$ for each feature variable-*i* is calculated using Equations (4) and (5), respectively:

(4) $$\begin{array}{cc} devD_{i}=\frac{\left|D_{i}^{k}-WavgD_{i}^{k-1}\right|}{WstdD_{i}^{k-1}} & \;\;\;\;for\;\;\eta \leq k\leq CCs, \end{array}$$

(5) $$\begin{array}{cc} tolD_{i}=|NORM.S.INV\left(\frac{1}{4*k}\right).| & \;\;\;\;for\;\;\eta \leq k\leq CCs. \end{array}$$

Here, NORM.S.INV indicates the inverse of standard normal cumulative distribution and CCs represents the total number of cardiac cycles. According to the empirical rule of statistics, in Gaussian distribution, 95% of data lies within two standard deviations from the mean and hence as per the thumb rule, one should consider no more than 5% of data as outliers. Hence, the value of tolerance $tolD_{i}$ is calculated in such a way that for those feature-variables whose duration value deviates more than two standard deviations from the mean of the corresponding feature variable is considered as outliers.

### 4.4. Combined Analysis of ECG and SCG Signals

Once the detection of the various types of abnormal morphologies is concluded from ECG and SCG signals. The next step is to ascertain that the cardiac cycle under consideration is indeed abnormal. Since, mere the detection of abnormal morphologies does not mean the abnormal behavior of the cardiac cycle, a Naïve Bayes probabilistic model is designed to know how likely the cardiac cycle under consideration is to be abnormal. For the probabilistic model design, ECG feature set consisting of seven features is used, which is represented as $FV_{ecg}=\left\{\Theta X_{wv},\psi X_{wv},\psi RR_{inv}\right\}$. Here, X∈{P,QRS,T}, and Θ and ψ represents measured wave amplitude, and duration, respectively. Similarly, SCG feature set consisting of six features is used, which is represented as $FV_{scg}=\left\{\Pi^{MC,AO},\Pi^{AO,AC},\Pi^{MC,MO},\Pi^{AC,MO},\Pi^{RBE},\Pi^{RBF}\right\}$.

The conditional Naïve Bayes probability model is designed to classify each cardiac cycle into class *normal* or *abnormal*. Let us say that for the $k^{th}$ ECG cardiac cycle with feature set $FV_{ecg}^{k}=\left\{\Theta X_{wv}^{k},\psi X_{wv}^{k},\psi RR_{inv}^{k}\right\}$, the conditional probability for a cardiac cycle to be *normal* or *abnormal* can be defined as shown in Equation (6):

(6) $$\begin{array}{c} p\left(\varphi_{l}|FV_{ecg}^{k}\right)=\frac{p\left(\varphi_{l}\right)\times p\left(FV_{ecg}^{k}|\varphi_{l}\right)}{p(FV_{ecg}^{k})},\;where\;l\in \left\{1,2\right\}. \end{array}$$

Here, $\varphi_{l=1}$ and $\varphi_{l=2}$ represents output class normal and abnormal, respectively. The $p(\varphi_{l=1}|FV_{ecg}^{k})$ and $p(\varphi_{l=2}|FV_{ecg}^{k})$ represents the probability of $k^{th}$ cardiac cycle to be normal and abnormal, respectively, for a given ECG feature set $FV_{ecg}^{k}$. The $p(\varphi_{l}|FV_{ecg}^{k})$ can be rewritten as shown in Equation (7):

(7) $$\begin{array}{c} p\left(\varphi_{l}|FV_{ecg}^{k}\right)=p\left(\varphi_{l}|\Theta X_{wv}^{k},\psi X_{wv}^{k},\psi RR_{inv}^{k}\right). \end{array}$$

Under the Naïve Bayes conditional independence assumption, each feature $x_{i}\in FV_{ecg}^{k}$ is assumed conditionally independent to every other features $x_{j}\in FV_{ecg}^{k}$ for j≠i. Hence, Equation (7) can be simplified as follows:

(8) $$\begin{array}{cc} p(\varphi_{l}|\Theta X_{wv}^{k},\psi X_{wv}^{k},\psi RR_{inv}^{k}) & \propto p(\varphi_{l},\Theta X_{wv}^{k},\psi X_{wv}^{k},\psi RR_{inv}^{k})\\ & \propto p\left(\varphi_{l}\right)\times p\left(\Theta X_{wv}^{k}|\varphi_{l}\right)\times p\left(\psi X_{wv}^{k}|\varphi_{l}\right)\times p\left(\psi RR_{inv}^{k}|\varphi_{l}\right)\\ & \propto p\left(\varphi_{l}\right)\times \prod_{i=1}^{i=6}p\left(x_{i}|\varphi_{l}\right),\;where\;x_{i}\in FV_{ecg}^{k}\\ & =\Xi \times p\left(\varphi_{l}\right)\times \prod_{i=1}^{i=6}p\left(x_{i}|\varphi_{l}\right),\;Here,\;\Xi \;is\;a\;constant. \end{array}$$

The Naïve Bayes conditional probability model defined in Equation (8) can be transformed into classifier using the maximum a posteriori decision rule as follows:

(9) $$\begin{array}{c} \Gamma_{ecg}=\underset{l\in \{1,2\}}{\mathrm{arg max}}p\left(\varphi_{l}\right)\times \prod_{i=1}^{i=6}p\left(x_{i}|\varphi_{l}\right),\;where\;x_{i}\in FV_{ecg}^{k}. \end{array}$$

Similarly, the Naïve Bayes conditional probability classifier as shown in Equation (10) can be constructed for SCG using a set of seven SCG features:

(10) $$\begin{array}{c} \Gamma_{scg}=\underset{l\in \{1,2\}}{\mathrm{arg max}}p\left(\varphi_{l}\right)\times \prod_{i=1}^{i=7}p\left(y_{i}|\varphi_{l}\right),\;where\;y_{i}\in FV_{scg}^{k}. \end{array}$$

Here, $\Gamma_{ecg}$ and $\Gamma_{scg}$ is assigned with class label $\varphi_{l}$ for some *l* based on the maximum a posteriori probability. Using the probabilistic outcome of $\Gamma_{ecg}$ and $\Gamma_{scg}$, each ECG and SCG cardiac cycle is marked as normal (i.e., binary ’0’) or abnormal (i.e., binary ’1’):

(11) $$\begin{array}{c} J_{outcome}=CC_{ecg}^{k}\wedge CC_{scg}^{k}, \end{array}$$

(12) $$\begin{array}{c} CAI=\frac{\#\;of\;abnormal\;CCs\;in\;a\;group}{Total\;\#\;of\;CCs\;in\;a\;group}. \end{array}$$

As mentioned in earlier sections, abnormalities in ECG cycles do not necessarily indicate the underlying abnormal cardiac activities and therefore we look for the abnormalities in corresponding SCG cardiac cycles as well. For an ECG and corresponding SCG cardiac cycle, if only one of them is detected abnormal (e.g., binary ’1’) by proposed Naïve Bayes probabilistic classifier, then a conclusion is drawn that the cardiac cycle under consideration is more likely to be normal in nature (e.g., binary ’0’) and observed abnormal morphology in either ECG or SCG cardiac cycle is due to the external reasons such as noise. However, if both ECG as well as corresponding SCG cardiac cycles are simultaneously detected with abnormal morphology, then a conclusion is drawn that the cardiac cycle under consideration is indeed abnormal. Equation (11) acts as an additional measure to generate reliable outcomes in the presence of signal artifacts. For example, if one of the modalities (let us say ECG) outputs the abnormal cardiac cycle (e.g., binary 1) due to external signal artifacts, and the other one (let us say SCG) outputs as entirely normal (e.g., binary 0), then the concerned cardiac cycle is treated as normal ( e.g., 1 ∧ 0 = 0) to reduce the false positives and to avoid the mis-interpretation of results. The outcome of the combined analysis of ECG and SCG cardiac cycle can be calculated as shown in Equation (11). Here, $CC_{ecg}^{k}$ and $CC_{scg}^{k}$ represent individual outcomes of $k^{th}$ cardiac cycle of ECG and SCG, respectively. The $J_{outcome}$ represents the combined outcome. In addition, Table 3 shows all possibilities that arise in Equation (11). A new parameter called Cardiac Abnormality Index (CAI) is defined to represent the intensity of cardiac abnormal behavior. The CAI is defined as the number of abnormal cardiac cycles out of the total number of cardiac cycles in a group as shown in Equation (12). The higher the value of CAI, the higher the risk of CVD and vice versa. If CAI increases gradually over the period of time and crosses the predefined threshold value δ, an alert warning may be issued to the user to consult the cardiologist. It is to note that the value of δ can be determined in consultation with the cardiologists.

## 5. Performance Evaluation

In this section, first, we describe the methodology that we have adopted to evaluate the performance of proposed ECG and SCG feature point delineation mechanisms followed by corresponding results. Since our proposed combined cardiac anomaly detection mechanism is based on the investigation of various feature points of ECG and SCG signals, first we need to verify the accuracy of the proposed ECG and SCG feature point delineation mechanisms. Performance evaluation is carried out on 12,000 cardiac cycles of ECG and SCG collected from three normal (*N*) and two abnormal (AN) real subjects using our IRB license as described in Section 2.1.

### 5.1. Demographic Information

For each subject, an individual data file comprised of ECG and SCG signals in the form of sampled data points is generated as an output as part of data collection process. Total 20 subjects are recruited for the data collection purpose with an equal number of male and female subjects, i.e., *10 subjects per gender*. The demographic information of the subjects is summarized in Table 4. The average age of the subjects is 24.45 years and the age ranges from 21 through 30 years. The data collection for all the subjects is carried out in supine posture as presented in Figure 1. Out of 20 subjects, 12 subjects are found as normal and eight subjects are considered as abnormal due to their sedentary lifestyle. The average height, weight, and BMI (Body Mass Index) of subjects is 1.52 (m), 59 (kg) and 22.9, respectively. In addition, Table 4 shows an example of value of amplitude in mV out of thousands/millions of mV measurements for the reference purpose only. The inclusion criteria are presumably healthy adult subjects with no known cardiac conditions, equal number of male and female subjects with age ≥ 18; whereas the exclusion criteria are the inability to provide written consents, sample size less than 20 subjects. For each subject, data collection is carried out for total 15 min consisting of three sessions of 5 min each with 5 min of a break between the successive sessions. It is to be noted that the entire data collection process is thoroughly verified by approved by an ethical committee of Institution Review Board (IRB) of Gung Memorial Hospital (CGMH), Taiwan with IRB license number 104-6615B.

All of the 20 subjects are chosen for experiment purposes. Since manual annotation is a highly laborious process, we present results based on the case study of 20 subjects. However, without losing the generality, it is to be noted that 12,000 cardiac cycles in total are considered from the selected subjects for joint investigation, which are statistically sufficient enough to interpret the trend and to draw a conclusion. The sample traces are prepared by extracting 10 min of ECG and SCG recording from the total available recording of 15 min for each subject. During data collection, the signals are obtained from each subject in three intervals of 5 min each. Out of three intervals, the first two intervals of 5 min are taken for data analysis purpose. Since a big enough number of cardiac cycles are obtained from 10 min of recording, the analysis is carried out on two sets of 5 min recordings only. Table 5 presents the observed number of cardiac cycles (OCCs) of five subjects for reference purposes.

### 5.2. Performance Metrics and Outputs

In order to investigate the effectiveness of the proposed automatic ECG and SCG feature points mechanisms, first we need to generate reliable ECG and SCG reference feature points. To comply with the scientific evidence and for the rigorous and reliable evaluation, a laborious manual annotation approach is adopted. A cardiologist is involved in the study to generate the highly reliable reference feature points. In the case of conflicted opinions among the annotators, the feature points are either accepted or rejected based on the majority opinions (i.e., 2 out 3). To confirm the reliability of manual annotation, the Kohen’s kappa coefficient (κ) is calculated to statistically measure the inter-annotator agreement. The high value of κ=0.73 shows the substantial agreement. The manually annotated feature points are considered as candidate feature points represented as ManFeaturePts. These ManFeaturePts act as a reference and are compared with feature points selected by proposed mechanisms. The same sample traces are considered as input and processed using proposed ECG and SCG feature points delineation mechanisms for automatic annotation. Popular evaluation methods are used such as Precision, Recall and F−measure for performance evaluation purpose, as defined in Equations (13)–(15), respectively. Here, parameter ManFeaturePts represents the set of feature points that are manually annotated by expert cardiologists and parameter AutoFeaturePts represents the set of feature points that are automatically annotated by the proposed mechanisms. It is to be noted that the SCG signals are obtained from the Tricuspid valve site. Although the morphology of SCG signals obtained from different sites slightly varies from each other, the number of feature points, i.e., nine in a cardiac cycle remains same. The minor morphology specific changes in algorithms may ensure the similar results of feature point delineation and abnormality detection for a given patient:

(13) $$\begin{array}{c} Precision=\frac{\left|\{ManFeaturePts\}\cap \{AutoFeaturePts\}\right|}{\left|\{AutoFeaturePts\}\right|}, \end{array}$$

(14) $$\begin{array}{c} Recall=\frac{\left|\{ManFeaturePts\}\cap \{AutoFeaturePts\}\right|}{\left|\{ManFeaturePts\}\right|}, \end{array}$$

(15) $$\begin{array}{c} F-measure=2*\frac{Precision*Recall}{Precision+Recall}. \end{array}$$

The resultant outputs of the ECG feature point delineation mechanism are summarized in Table 6 and Table 8, whereas the resultant outputs of SCG feature points delineation mechanism are summarized in Table 7 and Table 9. Table 6 reports the total number of automatic feature points along with the corresponding total number of manual feature points for the ECG trace. Similarly, Table 7 reports the total number of automatic feature points with the corresponding total number of manual feature points of the SCG trace for different subjects. For each subject, the evaluation parameters Precision, Recall and F−measure are calculated separately for both ECG and SCG modalities using the output data reported in Table 6 and Table 7, respectively. In addition, Table 8 reports the mean error (ms) between the automatic and manual ECG feature points delineation along with onset and offset of waves in terms of mean±SD, whereas Table 9 reports the mean error (ms) between the automatic and manual SCG feature points delineation in terms of mean±SD. From Table 8, it is observed that the mean error for the set of onset $\left\{P_{onset},QRS_{onset},T_{onset}\right\}$ and offset points $\left\{P_{offset},QRS_{offset},T_{offset}\right\}$ is marginally more compared to that of other peaks {P,Q,R,S,T}. Table 8 and Table 9 show that the feature point *R* and AO has the least mean delineation error, respectively.

The Precision indicates the fraction of selected feature points that are relevant. In other words, Precision is a measure of result relevancy, i.e., *accuracy* and it describes the ability of the algorithm to select the relevant, i.e., *correct* feature points. The higher the value of Precision indicates the low false positive rate FPR. On the other hand, Recall indicates the fraction of correct feature points that are retrieved. The Recall shows the ability of the algorithm to return the positive results. The higher the value of Recall indicates the low false negative rate FNR. Finally, the F−measure is a single value performance indicator, which represents the harmonic mean of the Precision and the Recall. The higher the value of F−measure indicates the better accuracy of the algorithm.

Performance evaluation results of ECG and SCG feature points delineation mechanisms are shown in Table 10. From the performance results, it is clear that the proposed feature point delineation algorithm of ECG marginally performs better as indicated by better outcome of the average Precision, Recall and F−measure values. The results of the proposed SCG feature point delineation algorithm also show the efficient annotation as indicated by average Precision, Recall and F−measure value. It is to be noted that the ECG and the SCG feature point delineation mechanisms perform better for the normal subjects as compared to the abnormal one. There are varieties of reasons behind the reduced value of F−measure for the abnormal subjects such as missing feature points, abnormal morphology, overlapping of waves, etc. In addition to the aforementioned reasons, the presence of external human vibrations (noise) recorded by sensors also negatively contribute the results. The F−measure value for SCG modality can be improved marginally by pre-processing the SCG traces using the data smoothing techniques such as simple moving average, weighted moving average, etc.

In the proposed ECG and SCG feature point delineation mechanisms, firstly, feature point *R* and AO is selected, respectively, in each cardiac cycle. The rest of the feature points are selected with respect to *R* in the case of ECG and AO in the case of SCG. Hence, the performance evaluation of *R* and AO is carried out under different values of threshold for $\sigma R_{pt}$ and $\sigma AO_{pt}$, respectively. The receiver operating curve (ROC) is obtained from the true positive rate (TPR) and false positive rate (FPR) of the ECG and SCG feature point delineation mechanisms in selecting *R* and AO, respectively. Figure 6a,b presents the output of ROC analysis for evaluation of *R* and AO under various threshold limits of $\sigma R_{pt}$ and $\sigma AO_{pt}$, respectively. In ROC analysis, the diagonal line shown as dotted black line in Figure 6a,b represents the random guess, and an area below the diagonal line represents the poor performance of the algorithm. The larger the area covered under the ROC curve, the better the performance of the algorithm. It can be seen from Figure 6a,b that the performance of delineation of feature point *R* and AO is at a maximum for $\sigma R_{pt}=0.7$ and $\sigma AO_{pt}=0.75$, respectively.

## 6. Results and Discussion

**Figure 9:**
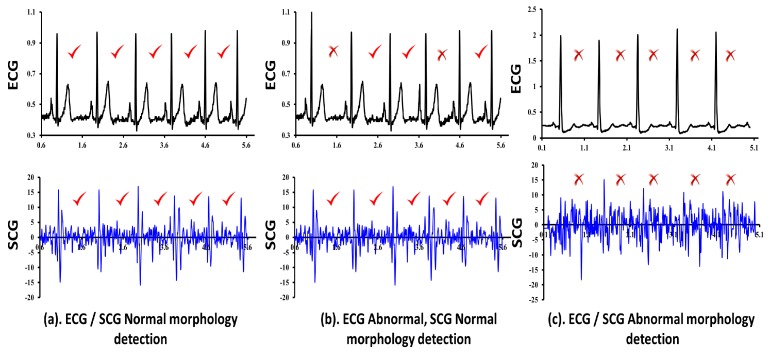
Combined evaluation of ECG and SCG signals using set of five cardiac cycles.

We have experimented with ECG/SCG traces of three normal and two abnormal subjects. The experiments are performed considering cardiac cycles in the group of 5-cycles, 10-cycles, 25-cycles and 50-cycles to verify the effectiveness of CAI. Based on our observations, CAI performs effectively well for the small sized group of cycles such as 5-cycles and 10-cycles for patients having chronic heart disease. In patients with chronic heart disease, abnormal cardiac cycles appear more frequently and the distance between two abnormal cardiac cycles is expected to be less as shown in Figure 9b,c. However, the effectiveness of CAI decreases drastically for small sized groups of cycles, in patients with occasional heart problems. In the case of occasional heart problems, it is less likely that an abnormal cardiac cycle appears frequently and together. Hence, most small sized groups of cardiac cycles output lower CAI leading to conclude that patient is normal, which is misleading. On the other hand, increasing the group size to 100-cycles and 200-cycles keeps the ratio of the number of abnormal cycles to the total number of cycles in a group very small, which also indicates the normal functioning of heart leading to the wrong diagnosis. Hence, in order to balance the effectiveness of CAI between the small sized groups and large sized groups, we experimented with medium sized groups such as 25-cycles and 50-cycles.

The sample output of combined cardiac anomaly detection for ECG and SCG considering the group of five cycles is shown in Figure 9. Three separate cases i.e., two for the normal subjects and one for an abnormal subject are shown for the group size of 5-cycles each. The first case for the normal subject is shown in Figure 9a, which shows the normal morphology detection in ECG as well as SCG cycles (CAI=0) and therefore we may conclude that there are no cardiac abnormalities in patient’s heart functioning, and he/she can be considered as normal. The second case for the normal subject is shown in Figure 9b, where two out of five ECG cardiac cycles are detected abnormal, one due to *R* amplitude abnormality and another due to *P* amplitude abnormality with CAI=0.4. However, the corresponding SCG cardiac cycles show no abnormalities with CAI=0 and therefore we may conclude that detection of abnormalities in ECG might be due to external noises. The third case for an abnormal subject is shown in Figure 9c, which shows the abnormal functioning of heart since all of the five ECG, as well as SCG cardiac cycles, have been recorded abnormal with CAI=1. Here, for ECG, the amplitude of *R* shows the significantly abnormal behavior in each cardiac cycle leading to the abnormality detection. On the other hand, corresponding SCG cardiac cycles also recorded as abnormal. Since cardiac cycles of both traces have simultaneously resulted in abnormal behavior, we may conclude that the patient has serious heart malfunctioning and he/she should immediately take the advice of cardiac experts.

**Figure 10:**
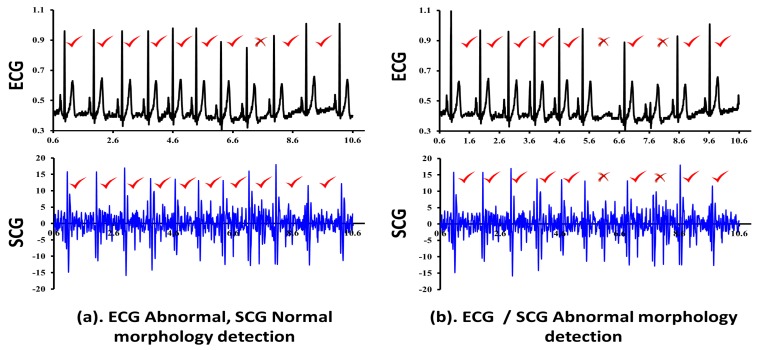
Combined evaluation of ECG and SCG signals using a set of 10 cardiac cycles.

The same experiments are repeated with the same set of input data considering the group sizes of 10-cycles, 25-cycles, 50-cycles, and 200-cycles. The experimental results for the group size of 10-cycles for normal subjects are shown in Figure 10, which shows the lower CAI values 0.1 and 0.2. In addition, we have also recorded the Timestamp instances of various feature points of ECG as well as the SCG traces. Table 11 shows the example Timestamp instances for the ECG feature points *P*, *Q*, *R*, *S* and *T*, whereas Table 12 shows the example Timestamp instances for the SCG feature points AS, MC, IM, AO, IC, RE, AC, MO and RF across two cardiac cycles for reference purposes only. From the two example cardiac cycles shown in Table 11, it is observed that the difference between successive Timestamp instances of ECG feature points are more consistent and regular for the normal subjects $A_{1}$, $A_{2}$ and $A_{3}$ than those observed for the abnormal subjects $A_{4}$ and $A_{5}$. Similarly, Table 12 reports the consistent behavior of SCG feature points for the normal subjects $A_{1}$, $A_{2}$ and $A_{3}$ than the abnormal subjects $A_{4}$ and $A_{5}$. The aforementioned observations do not specify the general conclusions, but state with respect to the randomly selected two examples of cardiac cycles. It is to be noted that one or more SCG feature points may not be repeated between two heartbeats due to external noise. For example, for subject-1 $\left(A_{1}\right)$ in Table 5, the reported number of cardiac cycles (defined as OCCs) is 825 and therefore the number of times AS and RF should appear is 825 times each. However, as reported in Table 7, the total number of manual feature points AS and RF those are retrieved for subject-1 is 791 and 801 times, respectively. This confirms the non-consistent repetition of AS and RF. The similar trend is observed for the rest of the subjects except abnormal subject 4 $\left(A_{4}\right)$ due to its highly distorted morphology. Due to the aforementioned limitations, the current study has considered analyzing the group of cardiac cycles together to capture the abnormal behavior of heart instead of a single cardiac cycle at a time.

The performance of any cardiac health monitoring system depends on its ability to accurately distinguish between normal and abnormal cardiac cycles. Hence, the performance of the proposed combined analysis of ECG and SCG is compared with the standalone use of ECG and SCG. All of the 12,000 cardiac cycles are chosen and equally divided into 12 sets of 1000 cardiac cycles each. For each set, the ability of the system to detect the number of *normal* and *abnormal* cardiac cycles is measured. At first, the baseline reference performance output is generated by recruiting expert cardiologist, who are tasked to manually distinguish the cardiac cycles into *normal* and *abnormal* for each set. Later, the performance of systems such as *ECG only*, *SCG only*, and *ECG and SCG* are compared with the baseline output.

**Figure 11:**
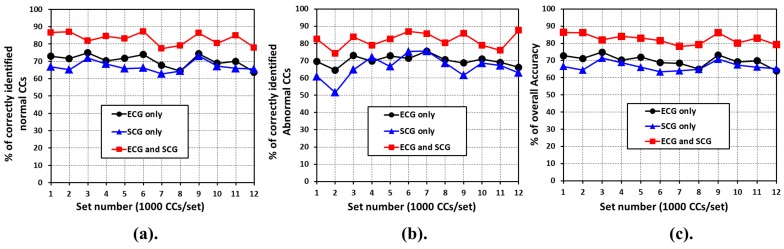
Performance comparison of ECG only, SCG only, and ECG and SCG combined analysis.

Figure 11 shows the output of each of the aforementioned systems with respect to the baseline performance. Figure 11a,b shows the comparison with respect to the ability of the proposed mechanisms to correctly detect the percentage of *normal* and *abnormal* cardiac cycles, respectively. As shown in Figure 11a,b, the combined analysis of ECG and SCG consistently outperforms the *ECG only* and *SCG only* mechanism. In addition, the performance of *ECG only* and *SCG only* is nearly identical to each other, with *ECG only* marginally performs better. The potential rational behind marginal improvement of ECG over SCG is due to the preprocessing of ECG signals for baseline wandering removal and noise reduction. The main reason behind the better performance of a combined analysis of *ECG and SCG* lies in its ability to validate and re-validate the *normal* and *abnormal* behavior of the cardiac cycle using cardiac electrical and mechanical information altogether. Figure 11c presents the performance of the systems with respect to the overall accuracy. As shown in Figure 11c, the combined analysis of *ECG and SCG* gives better overall accuracy as compared to the *ECG only* and *SCG only*.

## 7. Conclusions

In this paper, a combined cardiac health monitoring approach is presented using concurrent Electrocardiogram (ECG) and Seismocardiogram (SCG) signals. Each ECG and SCG cardiac cycle is tracked, analyzed and jointly investigated to distinguish the Normal and Abnormal behavior. A separate delineation mechanism is designed for the automatic delineation of specific feature points of ECG and SCG from the corresponding signal morphology followed by feature points based abnormal morphology detection mechanisms. For the reliable beat by beat analysis, individually detected abnormal morphologies in ECG and SCG are jointly investigated by designing a novel Naïve Bayes conditional probability model. The effectiveness of the proposed feature point delineation mechanisms is extensively verified against the performance metrics such as Precision, Recall, and F−measure by acquiring 12,000 concurrent ECG and SCG cardiac cycles from 20 real subjects approved by Institutional Review Board (IRB) of Chang Gung Memorial Hospital, Taiwan. Experimental results show that combined analysis of ECG and SCG can mitigate the disadvantages of both modalities up to some extent and can improve the cardiac health monitoring accuracy subject to the accurate feature points delineation. The main objective of the current study was to show that there is a scope for further improvement in continuous cardiac health monitoring, which can be accomplished by jointly analyzing the mechanical and electrical aspects of the cardiac activities obtained from SCG and ECG, respectively. The study is a step forward and needs to be continued for realizing the ultimate goal. The results presented in this study are encouraging and provide a solid foundation to explore the benefits of the combined investigation of ECG and SCG for future works. However, there are few limitations of this preliminary study. The current study only covers the basic ECG and SCG features. It could be possible that the better set of ECG and SCG features may further improve the results. In addition, the naive approach for the ECG delineation is employed, which can be further improved by using the proprietary algorithms designed to specifically delineate the ECG signal. The current combined analysis of one-lead ECG signals with concurrent SCG can be further extended and improved in the future work by engaging the two-lead ECG signals with concurrent SCG, as the two-lead ECG may provide more redundant information. In addition, the research can also be extended in the future by considering the non-detection of certain existing features as new features and incorporating them in machine learning algorithms to improve the accuracy of cardiac monitoring. The future work will include more ambulatory monitoring to test the algorithms in real conditions, i.e., with more noise and range of activities. It is to be noted that the results are obtained under a specific in-laboratory environment for subjects with specified demographic condition, and, therefore, results should be interpreted accordingly.

## Figures and Tables

**Table 1:** Notations for set of referenced normal feature values (RFV).

Notation	Meaning
$\Delta X_{wv}$	Referenced maximum *X* wave duration
$\delta X_{wv}$	Referenced minimum *X* wave duration
$\Delta Y_{inv}$	Referenced maximum *Y* interval duration
$\delta Y_{inv}$	Referenced minimum *Y* interval duration
$\Delta Z_{seg}$	Referenced maximum *Z* segment duration
$\delta Z_{seg}$	Referenced minimum *Z* segment duration
$\Omega X_{wv}$	Referenced maximum *X* wave amplitude
$\omega P_{wv}$	Referenced minimum *X* wave amplitude

Here, X∈{P,QRS,T}, Y∈{RR,PR,QT}, Z∈{PR,ST}.

**Table 2:** Notation of SCG feature-variables.

Notation	Meaning
$\Pi^{MC,AO}$	Time Duration from closing of mitral to opening of aortic.
$\Pi^{AO,AC}$	Time duration between opening and closing of aortic.
$\Pi^{MC,MO}$	Time duration between closing and opening of mitral.
$\Pi^{AC,MO}$	Time duration from closing of aortic to opening of mitral.
$\Pi^{RBE}$	Time duration of ventricle blood ejection.
$\Pi^{RBF}$	Time duration of diastolic blood filling.

$FV_{scg}=\left\{\Pi^{MC,AO},\Pi^{AO,AC},\Pi^{MC,MO},\Pi^{AC,MO},\Pi^{RBE},\Pi^{RBF}\right\}$.

**Table 3:** Combined analysis outcomes of ECG and SCG cardiac cycles.

${\mathrm{CC}}_{\mathrm{ecg}}^{k}$	${\mathrm{CC}}_{\mathrm{scg}}^{k}$	$J_{\mathrm{outcome}}$
0	0	0
0	1	0
1	0	0
1	1	1

Here, 0 = normal, 1 = abnormal.

**Table 4:** Demographic information of the subjects.

Subject No.	Gender	Age	Height	Weight	BMI	Posture	Lifestyle	ECG	SCG Tricuspid
			(m)	(Kg)				(mV)	(mV)
**1**	Female	28	1.69	66	23.1	Supine	Healthy	0.33	−4.98
**2**	Male	24	1.8	78	24.1	Supine	Sedentary	0.62	−6.49
**3**	Female	27	1.66	57	20.7	Supine	Healthy	0.22	−8.63
**...**	...	...	...	...	...	...	...	...	...
**20**	Male	23	1.71	62	21.2	Supine	Healthy	0.23	−1.08

**Table 5:** Sample ECG and SCG trace of five subjects with observed number of cardiac cycles (OCCs).

Type	Subject No	OCCs
**N**	**Subject-1 (**$A_{1}$**)**	825
**N**	**Subject-2 (**$A_{2}$**)**	638
**N**	**Subject-3 (**$A_{3}$**)**	712
**AN**	**Subject-4 (**$A_{4}$**)**	541
**AN**	**Subject-5 (**$A_{5}$**)**	527

**N** = Normal, **AN** = Abnormal.

**Table 6:** Sample result of ECG feature point delineation mechanisms.

Subjects (*A*)	ECG Feature Points											
	Total # of Automatic Feature Points						Total # of Manual Feature Points					
	*P*	*Q*	*R*	*S*	*T*	Total	*P*	*Q*	*R*	*S*	*T*	Total
*A*_1_	863	839	836	835	823	**4196**	819	822	825	821	818	**4105**
*A*_2_	666	649	640	653	629	**3237**	631	627	630	626	621	**3135**
*A*_3_	701	721	715	740	719	**3602**	708	710	712	709	711	**3550**
*A*_4_	609	608	589	616	609	**3031**	497	503	478	523	481	**2482**
*A*_5_	568	593	561	593	596	**2911**	489	490	493	529	510	**2511**

**Table 7:** Sample result of SCG feature point delineation mechanisms.

*A*	SCG Feature Points																			
	Total # of Automatic Feature Points										Total # of Manual Feature Points									
	AS	MC	IM	AO	IC	RE	AC	MO	RF	Total	AS	MC	IM	AO	IC	RE	AC	MO	RF	Total
*A*_1_	859	874	862	851	847	845	839	840	841	**7658**	791	786	779	816	793	787	811	809	801	**7173**
*A*_2_	668	653	648	642	651	659	666	653	663	**5903**	599	580	597	621	608	597	594	609	612	**5417**
*A*_3_	751	746	742	728	748	742	758	765	754	**6734**	667	691	681	703	667	661	670	659	680	**6079**
*A*_4_	600	611	595	581	577	598	603	581	590	**5336**	583	590	599	610	621	597	610	598	611	**5419**
*A*_5_	498	519	496	502	483	490	481	500	502	**4471**	455	519	501	481	452	493	481	477	468	**4327**

**Table 8:** The mean error (ms) between automatic and manual ECG feature points’ delineation.

b	*P*	*Q*	*R*	*S*	*T*	*P_onset_*	*P_offset_*	*QRS_onset_*	*QRS_offset_*	*T_onset_*	*T_offset_*
mean	1.4	2.7	0.6	1.1	1.7	2.4	2.1	2.3	2.0	2.5	2.8
± SD	2.1	1.8	1.0	1.3	0.9	1.4	1.1	1.6	1.5	1.7	2.1

**Table 9:** The mean error (ms) between automatic and manual SCG feature points’ delineation.

SCG Feature Point	*AS*	*MC*	*IM*	*AO*	*IC*	*RE*	*AC*	*MO*	*RF*
mean	1.04	1.41	1.78	0.37	1.49	1.22	1.30	1.34	1.10
± SD	0.7	0.5	0.8	0.7	0.6	0.8	0.4	0.7	0.6

**Table 10:** Performance evaluation of ECG and SCG feature points delineation mechanisms considering all 20 subjects.

*A*	For ECG			For SCG		
	Precision	Recall	F-measure	Precision	Recall	F-measure
$A_{1}$	0.978	0.995	0.986	0.936	0.966	0.951
$A_{2}$	0.968	0.982	0.975	0.917	0.943	0.93
$A_{3}$	0.985	0.997	0.99	0.902	0.948	0.925
$A_{4}$	0.819	0.917	0.87	0.84	0.92	0.88
$A_{5}$	0.86	0.95	0.91	0.80	0.91	0.85
**...**	...	...	...	...	...	...
$A_{20}$	0.925	0.87	0.90	0.84	0.87	0.85
**Avg**	**0.90**	**0.814**	**0.854**	**0.79**	**0.84**	**0.82**

**Table 11:** Delineation of ECG feature points with corresponding time instances.

Subjects (*A*)	ECG Feature Points									
	Cardiac Cycle-1					Cardiac Cycle-2				
	*P*	*Q*	*R*	*S*	*T*	*P*	*Q*	*R*	*S*	*T*
*A*_1_	0.338	0.453	0.484	0.50	0.75	1.313	1.43	1.46	1.48	1.73
*A*_2_	0.342	0.43	0.46	0.482	0.68	1.27	1.36	1.39	1.414	1.607
*A*_3_	0.46	0.58	0.61	0.631	0.854	1.3	1.421	1.45	1.472	1.701
*A*_4_	0.41	0.49	0.51	0.54	0.773	1.29	1.42	1.45	1.50	1.68
*A*_5_	0.38	0.53	0.56	0.611	0.83	1.356	1.49	1.52	1.56	1.78

**Table 12:** Delineation of SCG feature points with corresponding time instances.

*A*	SCG Feature Points																	
	Cardiac Cycle-1									Cardiac Cycle-2								
	*AS*	*MC*	*IM*	*AO*	*IC*	*RE*	*AC*	*MO*	*RF*	*AS*	*MC*	*IM*	*AO*	*IC*	*RE*	*AC*	*MO*	*RF*
*A*_1_	0.41	0.48	0.51	0.54	0.58	0.61	0.73	0.76	0.83	1.38	1.47	1.49	1.515	1.56	1.59	1.70	1.73	1.78
*A*_2_	0.57	0.62	0.64	0.66	0.71	0.77	0.87	0.9	0.99	1.43	1.465	1.49	1.51	1.54	1.6	1.68	1.74	1.82
*A*_3_	0.36	0.41	0.43	0.46	0.48	0.55	0.67	0.7	0.82	1.26	1.28	1.3	1.33	1.37	1.42	1.54	1.57	1.65
*A*_4_	0.52	0.593	0.622	0.655	0.701	0.73	0.87	0.9	0.99	1.58	1.651	1.68	1.713	1.767	1.8	1.941	1.97	2.06
*A*_5_	0.46	0.55	0.59	0.62	0.67	0.7	0.86	0.91	1.02	1.51	1.603	1.64	1.682	1.74	1.78	1.92	1.96	2.05

**A** = Set of subjects.
